# Prospective study of the efficacy of PCNL under local anesthesia based on the ERAS concept

**DOI:** 10.3389/fsurg.2025.1595466

**Published:** 2025-05-09

**Authors:** Zhaorong Liu, Longfei Yang, Jianbiao Huang, Dingyi Zhang, Yugen Li, Xiaoning Wang, Fengzhen Luo, Zhihua He

**Affiliations:** ^1^ Department of Urology, Yudu County People’s Hospital, Yudu, Jiangxi, China; ^2^ Department of Urology, The First Affiliated Hospital, Gannan Medical University, Ganzhou, Jiangxi, China; ^3^ Department of Urology, Jiangxi Cancer Hospital, Nanchang, Jiangxi, China; ^4^ Department of Urology, Zhongshan Hospital Xiamen University, School of Medicine, Xiamen University, Xiamen, China

**Keywords:** percutaneous nephrolithotomy, rapid recovery surgery, perioperative management, local anesthesia, surgical stress reaction

## Abstract

**Objective:**

To evaluate the feasibility, safety, and efficacy of local anesthesia applied to percutaneous nephrolithotomy (PCNL) under Enhanced Recovery After Surgery (ERAS) for treating upper urinary tract stones.

**Materials and methods:**

This study was a prospective, single-center randomized controlled study in which the patients were randomly divided into two groups: 40 in the ERAS PCNL under local anesthesia (ERAS-LA) group and 40 in the ERAS PCNL under general anesthesia (ERAS-GA) group). The primary indicators were stone-free rate; the secondary outcomes were intraoperative and postoperative complications, intraoperative and postoperative VAS pain scores and postoperative stress response indicators. A meta-analysis was also performed using RevMan 5.4 software by searching relevant literatures in PubMed/Medline, Web of Science and Embase.

**Results:**

The stone clearance rates at 48 h were similar between the two groups [ERAS-LA: 85.0% (34/40) vs. ERAS-GA: 87.5% (35/40), *P* = 0.800] and both 90% at 1 month. The incidence of surgical complications was similar between the two group. The intraoperative pain score in ERAS-LA group was 2.90 ± 0.74, and the postoperative 24-h pain score was comparable between the two groups (ERAS-LA: 2.65 ± 1.35 vs. ERAS-GA: 2.63 ± 0.98, *P* = 0.925), with good pain control. The mean total operative time was lower in ERAS-LA group than in ERAS-GA group (68.15 ± 24.11 min vs. 82.125 ± 20.42 min, *P* = 0.006). Postoperative hemoglobin change values (3.38 ± 3.00 × 10^9^/L vs. 5.22 ± 4.18 × 10^9^/L, *P* = 0.027) and stress response factors including C-reactive protein (8.39 ± 7.46 mg/L vs. 10.47 ± 10.30 mg/L, *P* = 0.035) and interleukin-6 (5.40 ± 1.50 pg/ml vs. 10.57 ± 1.82 pg/ml, *P* = 0.041) were significantly lower in ERAS-LA group. The mean catheter retention, fistula retention, and postoperative hospital stay were all significantly lower in ERSA-LA group than in ERSA-GA group (2.3%, 2.9%, and 5.08 days vs. 3.33%, 4.38%, and 6.35 days, *P* < 0.05). The results of the meta-analysis were similar to that of our study.

**Conclusions:**

Local anesthesia applied to ERAS-managed PCNL have a comparable stone clearance rates and complication rates, and a faster postoperative recovery, lower surgical stress, length of stay, anesthesia costs and hospital costs than general anesthesia.

**Clinical Trial Registration:**

http://www.medresman.org.cn, identifier (ChiCTR2100045681).

## 1 Introduction

With the change in living habits of people, surrounding environment, and diet, the incidence of urinary stones is on the rise (1). Among them, the incidence of upper urinary tract stones is significantly higher than that of lower urinary tract. Upper urinary tract stones can cause pain, hematuria, infection, hydronephrosis, etc. (2). Currently, percutaneous nephrolithotomy (PCNL) is the preferred surgical treatment for complex and loaded upper urinary tract stones, with the advantages of high stone-free rate (73%–96%), low trauma, and fast postoperative recovery (3, 4). However, PCNL surgery inevitably brings trauma and causes corresponding complications (5). In response to the related complications, urologists have done a lot of clinical research and practice on the procedure and perioperative management of PCNL, such as the choice of anesthesia, perioperative management of ERAS, etc. (6, 7).

The concept of rapid recovery surgery as a new perioperative concept refers to the application of a series of optimized perioperative management measures with evidence-based medical evidence. These measures include preoperative precise assessment (such as nutritional status optimization and infection control), intraoperative minimally invasive techniques (such as precise puncture and minimizing tissue damage), multimodal analgesia (local anesthesia infiltration combined with non-steroidal anti-inflammatory drugs), goal-oriented fluid management, and early postoperative mobilization. The aim is to mitigate the psychological and physiological stress responses of perioperative patients, thereby reducing postoperative complications and facilitating rapid recovery (5, 8). The application of ERAS in urology has also been reported, for instance, in radical cystectomy (9), radical prostatectomy (10), PCNL (7), etc. This approach has demonstrated favorable economic and social benefits and merits clinical promotion and application. Therefore, we aim to further investigate the impact of anesthesia selection on patients undergoing PCNL under ERAS perioperative management and identify more suitable ERAS model for PCNL.

In this study, local anesthesia and general anesthesia were selected to be applied to patients undergoing PCNL surgery with the implementation of the ERAS concept for a clinical randomized controlled study, and the relevant literature was also searched, and meta-analysis was performed to evaluate the feasibility, safety, efficacy, and impact on postoperative recovery of PCNL under local anesthesia based on the ERAS concept.

## 2 Materials and methods

### 2.1 Study design and patients

#### 2.1.1 Ethics and consent

The clinical study was a single-blind, randomized controlled study. It was reviewed and approved by the Ethics Committee of the First Affiliated Hospital of Gannan Medical College (No. LLSC-2020081201) and executed after completing the China Clinical Trials Registry review batch registration (registration number ChiCTR2100045681).

#### 2.1.2 Patient information and patient management

**Figure 1:**
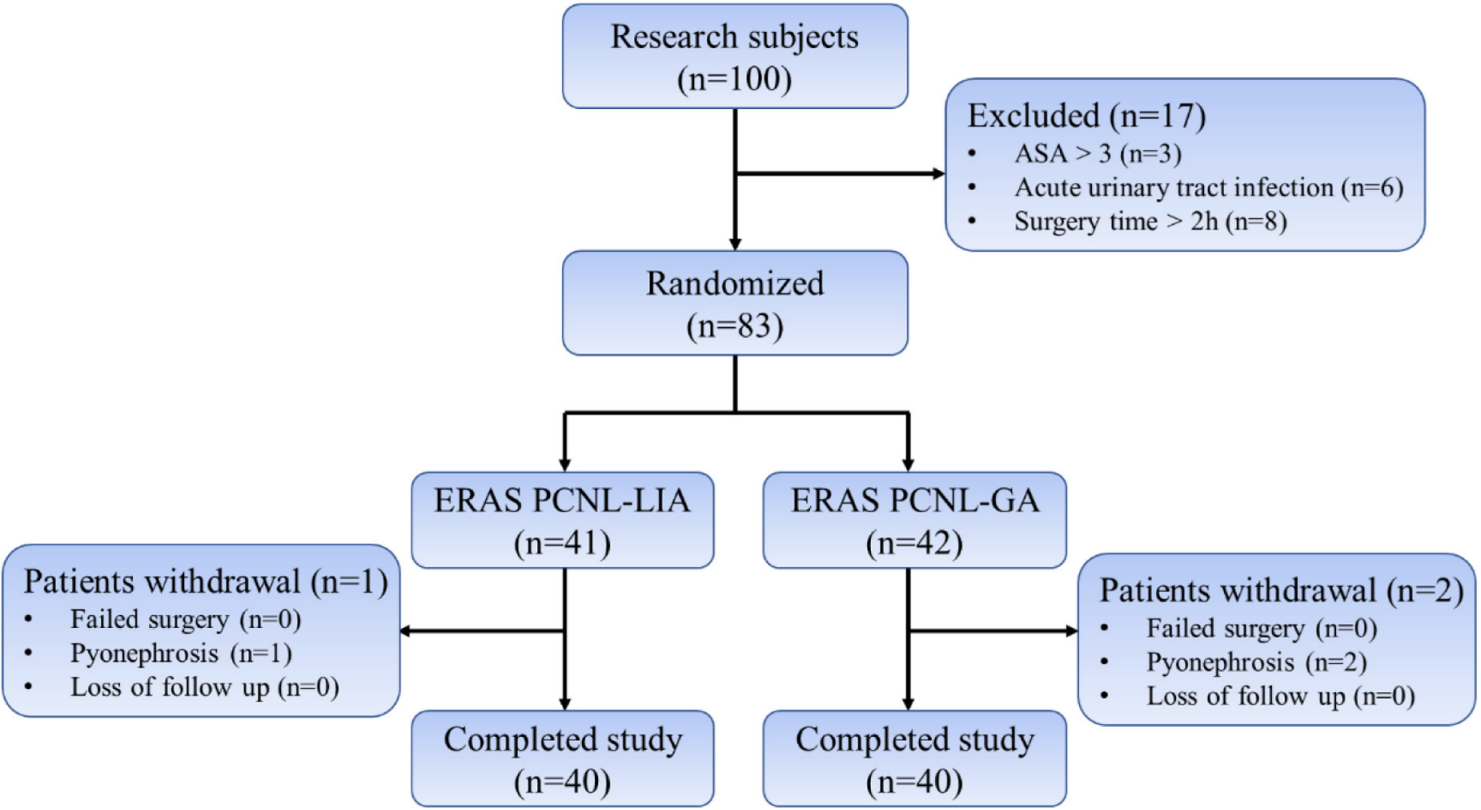
Flow chart.

A total of 100 patients admitted to the Department of Urology of the First Affiliated Hospital of Gannan Medical College between June 2019 and June 2021 for PCNL surgery after confirmed upper urinary tract stones were enrolled, of which 83 patients were randomly divided into the PCNL under local anesthesia group based on the ERAS concept (*n* = 41, ERAS-LA, experimental group) and under general anesthesia with ERAS group (*n* = 42, ERAS-GA, control group) (Figure 1) and patients in both groups underwent ERAS measures perioperatively (Table 1). The same senior title physician performed the PCNL procedures.

**Table 1:** Perioperative management of ERAS.

Measures	Local anesthesia ERAS	General anesthesia ERAS
Pre-operative education	Introduction to local anesthesia and ERAS concept and plan	Introduction to general anesthesia and ERAS concepts and plans
	Detailed information on preoperative preparation, specific procedures, and precautions for surgery	Detailed information on preoperative preparation, specific procedures, and precautions for surgery
	Perform psychological care to eliminate negative emotions such as anxiety, nervousness, and fear in patients and improve their confidence in the surgery. Instruct patients on postural training	Perform psychological care to eliminate negative emotions such as anxiety, nervousness, and fear in patients and improve their confidence in the surgery. Instruct patients on postural training
Pre-operative fasting	No special fasting or drinking is required before operation	6 h preoperative fasting, 2 h preoperative drinking, 2 h preoperative oral intake of 400 ml of “preoperative instant oral sugar”
Pre-anesthesia education	Emotional de-escalation by operating room nurses to ease discomfort and tension in the operating room	Emotional de-escalation by operating room nurses to ease discomfort and tension in the operating room
Intraoperative insulation Measures	1. The operating room temperature is maintained at 22℃–24℃. Humidity 50%–60%, maintain body temperature 36+℃ 2. Warming of infused and perfused fluids 3. Monitor the body temperature every half hour during the operation; if the body temperature drops, a heater blower is needed to keep it warm 4. Cover the quilt in time after operation	1. The operating room temperature is maintained at 22℃–24℃. Humidity 50%–60%, maintain body temperature 36+℃ 2. Warming of infused and perfused fluids 3. Monitor the body temperature every half hour during the operation, if the body temperature drops, a heater blower is needed to keep it warm 4. Cover the quilt in time after operation
Post-operative diet	No special postoperative abstinence from food and drink is required	2 h postoperative warm water mouth rinse, 6 h liquid diet
Post-operative activities	If there is no active hematuria after surgery, patients are encouraged to move around in bed but avoid strenuous and prolonged activities	After the patient is fully awake after surgery, if there is no active hematuria, encourage the patient to move around in bed but avoid strenuous and prolonged activities
Postoperative drainage tube	The nephrostomy tube may not be left in place after surgery according to the intraoperative situation, or the nephrostomy tube may be removed immediately after 24 h of surgery. According to the situation, the nephrostomy drainage tube may be removed on the third to fifth day after surgery. The urinary catheter may be removed or not left in place after surgery without significant bleeding	The nephrostomy tube may not be left in place after surgery according to the intraoperative situation, or the nephrostomy tube may be removed immediately after 24 h of surgery, and the nephrostomy drainage tube may be removed on the third to fifth day after surgery according to the situation, and the urinary catheter may be removed or not left in place after surgery without significant bleeding

#### 2.1.3 Inclusion and exclusion criteria

Inclusion criteria: (a) age between 18 and 80 years; (b) calcium stones, single pelvic stones <3.5 cm; (c) ureteral stones >1.5 cm above L4 level; (d) mild or above hydronephrosis; (e) no hepatic or renal dysfunction; no primary diseases of the hematopoietic system, cardiovascular, kidney, and liver, and ASA score less than 3; (f) informed consent obtained from patients and families.

Exclusion criteria: (a) Complex stones with a prognosis of >2 h; (b) Those who cannot cooperate with the position intraoperatively, including spinal deformity, poor pulmonary function, etc.; (c) Patients with uncontrolled acute urinary tract infection in combination with stones; (d) Intraoperative addition of skinned kidney access; (e) Second surgery in the same period.

#### 2.1.4 Random grouping and blinding

Patients were randomly assigned to the experimental and control groups using consecutive sealed opaque envelopes prepared by a third-party biostatistician using a random number table and double-blind method. The same subject physicians (unaware of the grouping) were then responsible for collecting patient data and assessing the patient condition.

### 2.2 Anesthesia and surgical methods

Local anesthesia group: preoperative 15–30 min intramuscular injection of “Dufay's combination” (pethidine hydrochloride injection 75 mg + promethazine hydrochloride 25 mg) for analgesia, and intraoperative 1% lidocaine for local anesthesia (11). A standardized PCNL procedure was performed. Briefly, patients were placed in a lithotomy position, and the urethra was perfused with lidocaine for 5 min before a 5F ureteral catheter was placed using a ureteroscope. After the catheter was left in place and the ureteral catheter was secured, the patient was converted to prone position on his own. According to the preoperative imaging examination, the direction of the puncture and the depth of needle entry was determined by combining ultrasound. 5 ml syringe (No. 5 needle) and 1% lidocaine subcutaneous infiltration anesthesia (2–3 cm in diameter) were used, and then the puncture path was anesthetized layer by layer to the perirenal area. Once ideal anesthesia was achieved, an 18G puncture needle was used to pierce the collecting system under ultrasound guidance, the needle core was withdrawn to place a zebra guidewire, and the skin was incised. The skin was incised to the deep fascial layer, which was sequentially dilated using a fascial dilator, and a 14–18F Peel-away working sheath was placed. The 8/9.8F ureteroscope was placed via the sheath to maintain adequate space for drainage between the skin and ureteroscope and to control intrarenal perfusion pressure <30 mmHg (12). Holmium laser (500 μm optical fiber, power 60 W) was used for lithotripsy. After lithotripsy, a 5F ureteral stent was placed anterograde. After subcutaneous infiltration anesthesia around the tube again, the procedure was concluded by leaving and suturing the nephrostomy tube (13, 14).

General anesthesia group: conventional endotracheal intubation method of general anesthesia was used. A standardized PCNL procedure was adopted for the surgical approach.

## 3 Outcome

### 3.1 Primary indicators

Postoperative stone-free rate: postoperative (within 48 h) review of KUB and, if necessary, urological CT, defined as stone residual by stone diameter >4 mm; return to the hospital 1 month after surgery for review of stone-free rate and removal of double J tube.

### 3.2 Secondary indicators

Post-operative hospital days: the time between the end of the operation and discharge from the hospital in compliance with the discharge criteria.

Assessment of bleeding volume: assessed according to the difference between preoperative and postoperative (within 24 h) hemoglobin.

Pain scoring: a visual analog pain score scale (VAS) was used that recorded patients' intraoperative and 24 h postoperative pain scores (15).

Stone load assessment: the S.T.O.N.E scoring system was used The preoperative stone load was assessed (16).

Postoperative complications grading status: the Clavien-Dindo grading system was used and graded into 5 grades (17).

### 3.3 Statistical analysis

SPSS 27.0 was applied to analyze the data. The measurement data were described by mean ± standard deviation (normal distribution) or median (non-normal distribution), and an independent sample *t*-test was used if the normal distribution was satisfied; otherwise, a non-parametric test was used, and two-way grouped ANOVA (two-way ANOVA) was used to compare data changes over time between multiple groups; the count data were expressed using frequency, and proportions were expressed using *χ*^2^ test or Fisher's exact probability method, correction formula, as appropriate; differences were statistically significant at *P* < 0.05.

### 3.4 Meta-analysis

**Figure 2:**
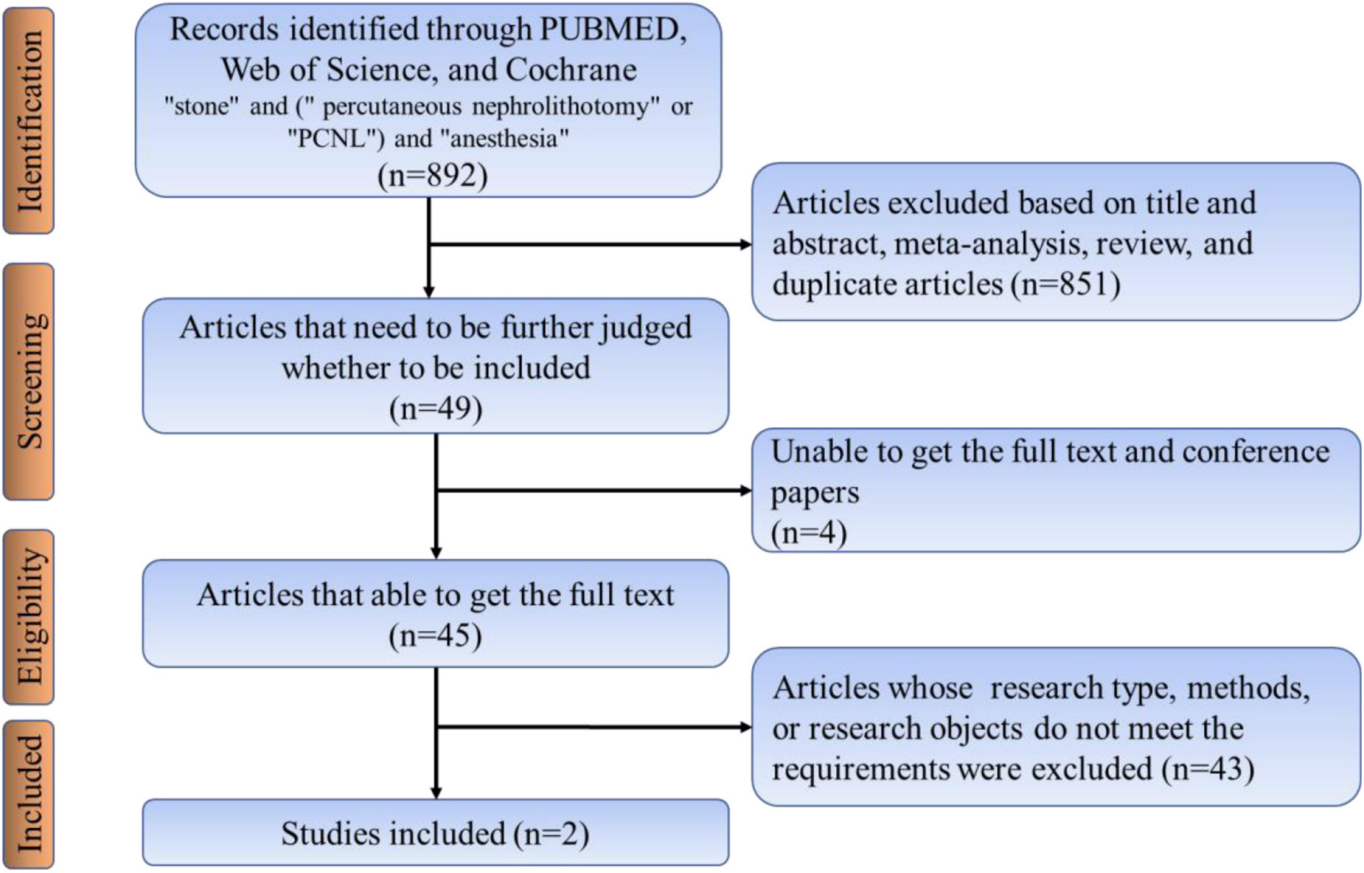
Flow chart of the articles retrieving.

Searches for relevant studies were performed by searching PubMed/Medline, Web of Science, Embase databases and the following search terms (searched March 2019, updated searches through October 2021): “stone” and (“percutaneous nephrolithotomy” or “PCNL”) and “anesthesia.” This study included all studies except Chinese, and all included articles strictly followed the nadir criteria. Inclusion criteria included:(1) Retrospective case-control studies, non-randomized controlled trials (nRCTs), and randomized controlled trials (RCTs). (2) Patients were required to have their stones removed using PCNL. (3) The study must include comparing local and general anesthesia. (4) Data from the included studies should be available for analysis. Exclusion criteria include patients with poor underlying conditions who cannot be included in the study. The literature search process was performed, as shown in Figure 2. Two authors (Liu and Zhang) independently screened and evaluated all retrieved citations and abstracts and followed Cochrane criteria to identify eligible studies for inclusion. Any disagreements about the studies were resolved by discussion with a third author.

## 4 Results

### 4.1 Baseline and perioperative data of patients

A total of 83 patients were randomly divided into the ERAS-LA group (*n* = 41) and the ERAS-GA group (*n* = 42), after excluding patients who underwent intraoperative abscess stage (1 in ERAS-LA group and 2 in ERAS-GA group), and finally, 40 patients were analyzed in each of the two groups. There were no statistical differences between the two groups regarding preoperative general condition, stone characteristics, and underlying disease (Table 2).

**Table 2:** Surgical characteristics of patients.

Projects	Local anesthesia group (*n* = 40)	General anesthesia group (*n* = 40)	t/*χ*^2^ value	*P*-value
Average age (years)	50.30 ± 11.40	52.50 ± 11.77	0.085	0.39
Average BMI (kg/m^2^)	23.37 ± 2.86	23.37 ± 2.86	1.311	0.19
Gender	–	–	0.053	0.818
Male	24 (60%)	25 (62.5%)	–	–
Women	16 (40%)	15 (37.5%)	–	–
Preoperative anxiety score	2.25 ± 0.59	2.18 ± 0.45	−0.689	0.493
Urine culture	–	–	0.581	0.446
Positive	12 (30%)	9 (22.5%)	–	–
Negative	28 (70%)	31 (77.5%)	–	–
Preoperative urine leukocytes	222.5 ± 355.77	200 ± 359.33	−0.154	0.878^a^
ASA Score	2.175 ± 0.26	2.075 ± 0.38	−1.351	0.181
Preoperative comorbidities	18 (45%)	15 (37.5%)	0.464	0.496
High blood pressure	9	8	0.75	1
Diabetes	4	2	–	0.675
Lung Diseases	1	1	–	1
Heart Disease	2	3	–	1
Coronary heart disease	1	0	–	–
Coronary stent implantation	1	0	–	–
Sinus bradycardia	0	2	–	–
Premature ventricular contractions	0	1	–	–
Chronic renal insufficiency	2	0	–	0.494
Obsolete cerebral infarction	0	1	–	1
S.T.O.N.E Rating	8.25 ± 1.46	7.98 ± 1.67	−0.783	0.436
CT value of stones (HU)	948.5 ± 312.33	978.30 ± 316.59	0.418	0.67
Stone diameter (mm)	21.6 ± 5.76	20.73 ± 5.227	−0.711	0.479
Surgical side	–	–	0.453	0.501
Left side	23 (57.5%)	20 (50%)	–	–
Right side	17 (42.5%)	20 (50%)	–	–
Stone location	–	–	5.407	0.062
Renal pelvis	22 (55%)	28 (70%)	–	–
Ureteral junction of the renal pelvis	2 (5%)	5 (12.5%)		
Ureter	16 (40%)	7 (17.5%)		

^a^Using rank sum test.

### 4.2 Comparison of intraoperative conditions

**Figure 3:**
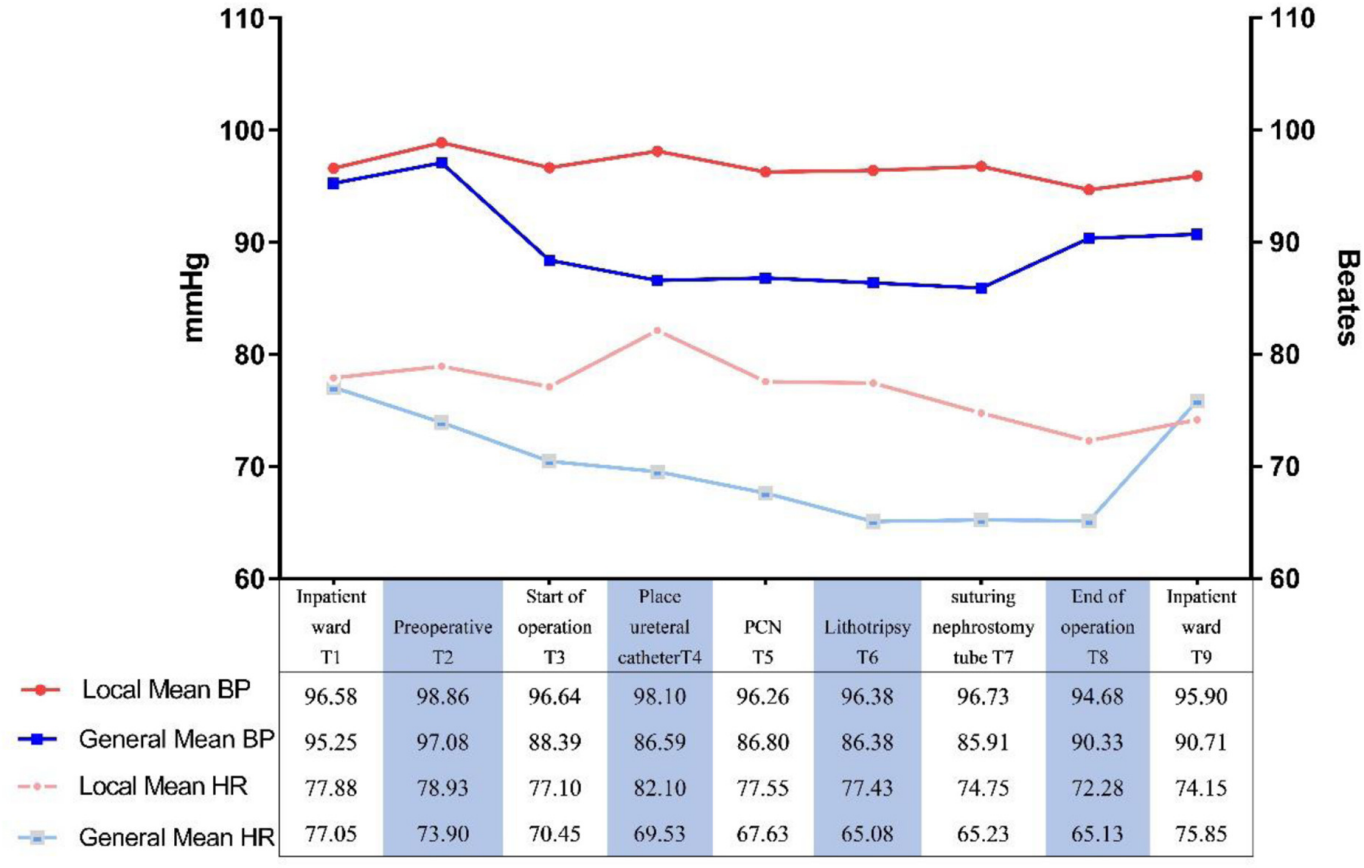
Comparison of intraoperative mean heart rates and mean blood pressure of groups. BP, blood pressure; HR, heart rate. **P < 0.01.

Local anesthesia group, while one case of sudden transient ventricular fibrillation and excessive bleeding requiring blood transfusion occurred in the general anesthesia group. The mean total operative time was lower in the local anesthesia group than in the general anesthesia group (68.15 ± 24.11 min vs. 82.125 ± 20.42 min, *P* = 0.006). Comparing the intraoperative vital signs of the two groups, the mean arterial pressure and heart rate in the local anesthesia group were higher than those in the general anesthesia group (96.68 mmHg 77 beats/min vs. 89.71 mmHg 70 beats/min; *P* < 0.01, *P* < 0.01). Preoperative (T1) mean arterial pressure and heart rate were defined as basal vital signs, and the intraoperative (T3) time points in the general anesthesia group showed statistically significant differences compared with basal blood pressure (*P* < 0.01) (Table 3; Figure 3).

**Table 3:** The incidence of intraoperative complications.

Projects	Local anesthesia group (*n* = 40)	General anesthesia group (*n* = 40)	χ2/t value	*P*-value
Intraoperative VAS score	2.90 ± 0.74	–	–	–
Intraoperative complications	0	2 (5%)	2.051	1
Blood Transfusion	0	1	–	–
Sudden transient ventricular fibrillation	0	1	–	–
Surgery time (min)	68.15 ± 24.11	82.125 ± 20.42	2.797	0.006*
Mean arterial pressure (mmHg)	96.68	89.71	2.499	0.001**

* *P* < 0.05.

** *P* < 0.01.

### 4.3 Postoperative results

Comparison of pain, use of special pain medication, complications, and incidence of infection complications at 24 h postoperatively was similar between the 2 groups. The stone-free rate at 48 h and 1 month postoperatively were identical between the 2 groups (Table 4). Postoperative renal function changed in both groups (*P* < 0.01), but the difference was not statistically significant when comparing renal function between the 2 groups preoperatively and postoperatively (*P* = 0.59, 0.71). The value of blood leukocyte change was lower in the local anesthesia group than in the general anesthesia group (3.38 ± 3.00 × 10^9^/L vs. 5.22 ± 4.18 × 10^9^/L, *P* = 0.027). Intraoperative and postoperative fluid replacement and hemoglobin loss were lower in the local anesthesia group compared to the general anesthesia group. The duration of catheter retention, fistula retention, and postoperative hospitalization in the local anesthesia group were (2.3, 2.9, and 5.08) days, respectively, which were lower than those in the general anesthesia group of (3.33, 4.38, and 6.35) days, respectively, and the difference was statistically significant (*P* < 0.05). Hospitalization costs were lower in the local anesthesia group than in the general anesthesia group when comparing the two groups.

**Table 4:** Postoperative condition.

Projects	Local anesthesia group (*n* = 40)	General anesthesia group (*n* = 40)	t/*χ*^2^ value	*P*-value
Stone free rate at 48 h after surgery	34 (85%)	35 (87.5%)	0.105	0.745
Stone-free rate at 1 month after surgery	36 (90%)	36 (90%)	–	1
Second stage surgery (same hospitalization)	1 (2.4%)	2 (4.8%)	–	1
Postoperative VAS score	2.65 ± 1.35	2.63 ± 0.98	−0.095	0.925
Special pain medication use (people)	13 (32.5%)	8 (20%)	1.614	0.204
Intraoperative fluid rehydration volume (ml)	532.5 ± 47.4	1,242.5 ± 280.9	15.762	0.00**
Postoperative 24 h rehydration volume (ml)	210 ± 44.14	1,790 ± 310.3	31.879	0.00**
Decrease in hemoglobin (g/L)	5.93 ± 6.9	10.18 ± 7.54	−2.420	0.01^a^*
Preoperative creatinine (μmol/L)	96.75 ± 44.00	92.25 ± 29.56	−0.537	0.59
Postoperative creatinine (μmol/L)	101.20 ± 49.64	97.83 ± 29.54	−0.37	0.71
Creatinine change value (μmol/L)	4.45 ± 13.74	5.57 ± 13.57	−7.47	0.455^a^
Postoperative and preoperative comparison between the two groups	–	–	−3.301	0.00**
Preoperative leukocytes (*10^9^/L)	7.15 ± 1.75	7.55 ± 2.30	0.883	0.38
Postoperative leukocytes (*10^9^/L)	10.52 ± 2.54	12.77 ± 4.04	2.973	0.004**
Leukocyte change values (*10^9^/L)	3.38 ± 3.00	5.22 ± 4.18	2.260	0.027*
Complications of infection	6 (15%)	9 (22.5%)	0.738	0.568
Fever	6	6	0	1
SIRS	0	3	–	0.241
Postoperative complications Clavien classification	8 (20%)	10 (25%)		0.92
I	6*	6*		1
Fever	6	6		–
vomiting	0	1		–
Hemostasis	2	1		–
II	0	1		1
Blood transfusion	0	1		–
III	2*	3*		1
Renal arteriography	0	1		–
Urine exosmosis, adjustment of double J tube	2	1		–
Lower extremity vein thrombosis	0	1		–
IV	0	0		0
Duration of postoperative catheter retention (days)	2.3 ± 1.87	3.33 ± 1.54	2.674	0.009**
Duration of postoperative fistula retention (days)	2.9 ± 2.04	4.38 ± 1.76	3.451	0.001**
Postoperative hospitalization days (days)	5.08 ± 2.69	6.35 ± 2.35	2.257	0.027**
Hospitalization cost (yuan)	15,662.88 ± 4,407.10	23,742.35 ± 9,706.39	4.794	0.000**
Anesthesia cost (yuan)	63.75 ± 119.84	2,123.05 ± 877.62	9.58	0.000**

^a^ Using rank sum test.

* *P* *<* *0*.05.

** *P* < 0.01.

### 4.4 Stress response indicators

**Figure 4:**
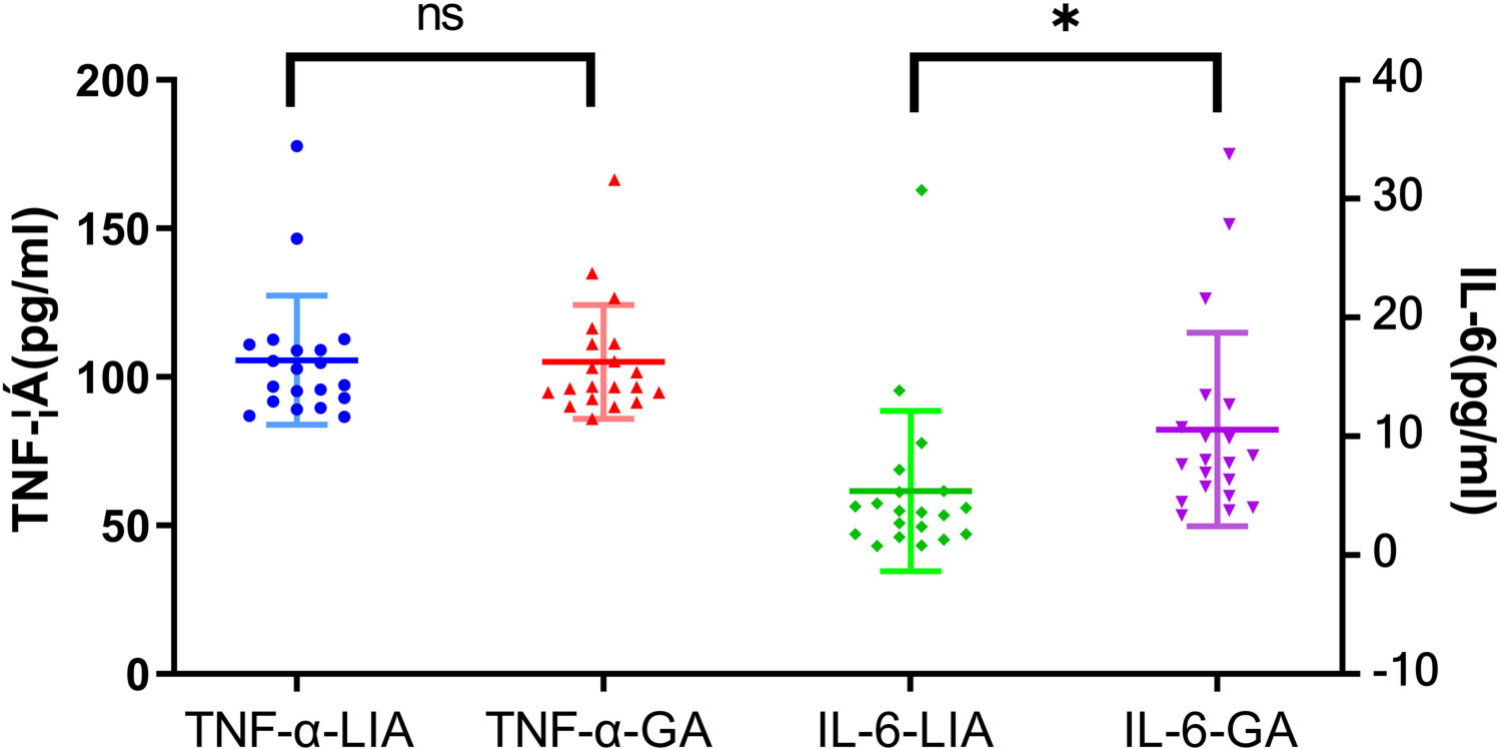
Postoperative tumor necrosis factor-α and interleukin-6 levels in two groups of patients. ^ns^ *P* > 0.05; *P < 0.05.

Postoperative C-reactive protein and interleukin-6 were lower in the local anesthesia group than in the general anesthesia group (8.39 ± 7.46 mg/L, 5.40 ± 1.50 pg/ml vs. 10.47 ± 10.30 mg/L, 10.57 ± 1.82 pg/ml, *P* = 0.035, 0.041) (Table 5; Figure 4).

**Table 5:** Changes in C-reactive protein.

Item (mg/L)	Local anesthesia group	General anesthesia group	*Z*-value	*P*-value
Preoperative C-reactive protein	3.43 ± 3.42	2.89 ± 2.82	−0.645	0.519
Postoperative C-reactive protein	8.39 ± 7.46	10.47 ± 10.30	−2.040	0.041*^,^^a^
Difference^d^	4.96 ± 6.10	7.58 ± 9.84	−3.440	0.001**
Pre-op—post-op comparison	–	–	−6.802	0.00**^,^^a^

^a^Using rank sum test.

^d^ Indicates the difference between preoperative and postoperative C-reactive protein.

* *P* *<* 0.05.

** *P* < 0.01; a using rank sum test.

### 4.5 Meta-analysis results

**Figure 5:**
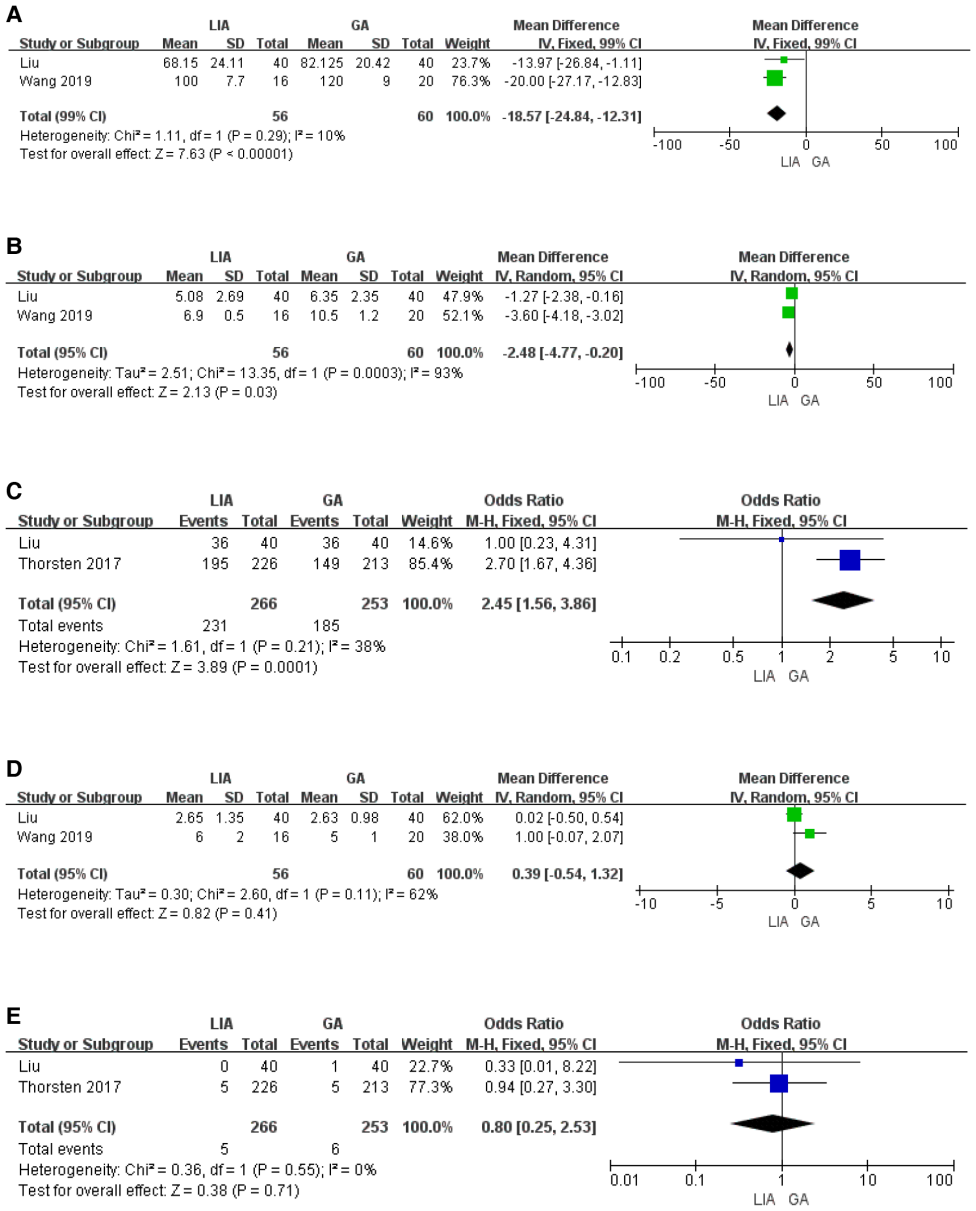
Results from the meta-analysis comparing the surgical effects of PCNL under local and general anesthesia. **(A)** Duration of operation. **(B)** Hospital stay. **(C)** Stone free rate. **(D)** VAS score. **(E)** Transfusion.

A search of published articles identified 898 results relevant to the search. After reading through the abstracts and excluding them, only 2 studies met the inclusion criteria. In the meta-analysis that included the results of our research, the local anesthesia group had less operation time and hospital stay compared to the general anesthesia group (Figures 5a,b). The risk of residual postoperative stones was greater in the general anesthesia group compared to the local anesthesia group (OR 2.45, 1.56–3.85) (Figure 5c). In contrast, the two groups had no significant difference in VAS score and transfusion rate (Figures 5d,e).

## 5 Discussion

In this study, a single-center RCT trial, combined with a meta-analysis of relevant literature, was conducted to evaluate the effect of local anesthesia applied to PCNL patients managed by ERAS, which can improve the prognosis of PCNL patients by minimizing perioperative irritation to reduce the surgical stress response of patients (18). Perioperative-related stimuli include damage to the immune barrier (surgical trauma, pain, etc.), invasion of pathogens, alterations in catabolism (fasting, etc.), and alterations in the systemic internal environment (anesthesia, bleeding, hypothermia, electrolyte balance disturbances, etc.).

Pain is a serious irritant that can cause various serious complications and affect the patient's recovery, and effective pain control is the basis for rapid recovery (19). General anesthesia is effective for pain control, but PCNL surgery under general anesthesia can easily lead to intraoperative complications such as hypotension and hypothermia. In this clinical study, the mean intraoperative VAS score of patients in the local anesthesia group was 2.9 (0.5–4.4, mild pain), and the postoperative pain score was comparable to that of the general anesthesia group, with good pain control. Moreover, the meta-analysis lent support to our findings. Intraoperative vital signs (mean arterial pressure, heart rate) were monitored and controlled within safe limits in both groups (80–100 mmHg, fluctuations ±20%) (20, 21). However, the degree of fluctuation of vital signs in the local anesthesia group was less than that in the general anesthesia group, which had less impact on the intraoperative circulatory system. The reasons for this may be related to the need to change the position during PCNL, a large amount of perfusion flushing fluid, the redistribution of blood by general anesthetic drugs, and the inhibition of the brain center, which affects the circulatory system (22, 23).

Patients under general anesthesia are sedated intraoperatively due to anesthetic drugs and cannot provide timely feedback on changes in body temperature and pain due to high intrarenal pressure and surgical trauma. Hypothermia affects platelet and prothrombin function (23, 24); the surgical trauma will destroy the immune barrier function of ureter and renal pelvis mucosa, increasing the risk of bleeding and infection; when combined with high intrarenal pelvic pressure, it will easily lead to reflux and urinary extravasation, increasing the occurrence of complications such as infection, bleeding and urogenic sepsis. In this study, the amount of postoperative hemoglobin loss and blood leukocyte changes in the local anesthesia group were better than those in the general anesthesia group, similar to the results of other studies (6). It may be related to the ability to stay awake during local anesthesia, and intraoperative wakefulness is beneficial for (1) timely response to renal pain, facilitating intrapelvic pressure control (<30 mmHg), and reducing the occurrence of infection complications, (2) timely reporting of unexpected surgical events (such as sudden pain, pleural injury, etc.) allows the attending physician to promptly adjust the renal access dilation depth or angle, preventing excessive manipulation and thereby reducing trauma; (3) timely reflection of body temperature changes, effectively preventing the occurrence of intraoperative hypothermia (6).

Perioperative fluid precision management is an important component of ERAS (25); excessive fluid rehydration is prone to complications such as pulmonary edema, heart failure, and gastrointestinal mucosal edema (26). Especially for elderly patients or those with cardiopulmonary diseases, whose cardiopulmonary reserve function decreases, fluid overload is more likely to induce acute left heart failure or respiratory insufficiency. Thus, the meticulous regulation of intraoperative fluid balance is of particular importance (27). In this study, both groups also adopted the ERAS concept to shorten the preoperative fasting time and encourage early postoperative activities, but the amount of fluid rehydration in the local anesthesia group was significantly lower than that in the general anesthesia group, and they were able to resume bed activities earlier after surgery. This may be related to the fact that general anesthesia inhibits the function of the gastrointestinal tract and necessitates preoperative and postoperative fasting and bed rest until full wakefulness (28, 29). This advantage may be even more evident in elderly patients or those with cardiopulmonary insufficiency. By reducing the fluid load, the risk of perioperative cardiovascular events can be mitigated, and early mobilization can further enhance the effectiveness of cardiopulmonary function rehabilitation in these patients. Prolonged fasting during the perioperative period can lead to insulin resistance and impaired gastrointestinal tract function, increasing postoperative metabolic stress (30). The local anesthesia group does not require special fasting and has low blood pressure fluctuations, so fluid management is simpler, and the patient can get out of bed earlier after surgery, shortening the time of catheter and nephrostomy tube placement and avoiding discomfort caused by the tube (30). This shortens the time of catheter and nephrostomy tube placement and avoids the discomfort of the activities of patients caused by the tubes. The early postoperative activity helps reduce complications such as insulin resistance and pulmonary infections, thus accelerating the recovery of body functions (5). Early postoperative activity can help reduce the complications of insulin resistance and pulmonary infection, thus accelerating the recovery of physical function.

The mechanism of the perioperative stress response is complex. Previous studies have demonstrated that its core pathways involve the cascade amplification of neuroendocrine activation and the systemic inflammatory response (31). Surgical trauma activates the spinal dorsal horn and cerebral cortex via afferent nerves, triggering the sympathetic-adrenal medulla system and the hypothalamus-pituitary-adrenal axis, leading to a substantial release of stress hormones, such as catecholamines and cortisol (31); On the other hand, the production of pro-inflammatory cytokines increases, including C-reactive protein (CRP), tumor necrosis factor-α (TNF-α), interleukin-6 (IL-6), etc. (32). In particular, IL-6 and CRP increase rapidly within 18–24 h and 48–72 h after surgery, respectively. These cytokines are produced in response to damage to numerous cells throughout the body and form a complex cascade of “neuro-endocrine-immune” interactions. In this study, the postoperative stress factors (C-reactive protein and interleukin-6) in the ERAS management group under local anesthesia were lower than those in the general anesthesia group; and the postoperative hospitalization time and cost were lower than those in the general anesthesia group, this is similar to the results of meta-analysis (6). It is tentatively suggested that applying local anesthesia to ERAS management can reduce the stress response of organism and thus promote the postoperative recovery of patients.

The present study demonstrates several key strengths alongside its limitations.

### 5.1 Strengths

1. ERAS-integrated anesthesia model: In contrast to previous research that focused solely on anesthesia techniques (such as nephrostomy tract infiltration), our protocol systematically integrates ERAS components—including prehabilitation, optimized fluid management, and multimodal analgesia—to assess the synergistic effects of local anesthesia within the ERAS framework, with the potential to establish a novel PCNL rehabilitation paradigm.
2. Comprehensive outcome assessment: Beyond traditional surgical success metrics (stone-free rate, complications), we incorporated inflammatory index (CRP, TNF-α, IL-6), economic indicators, and recovery outcomes (hospital length of stay), providing a multi-dimensional evaluation of local anesthesia's impact.
3. Enhanced research design: On the basis of prospective RCT studies with higher evidence level, the limitations caused by single center and small sample are minimized by adding value through Meta analysis.

### 5.2 Limitations

1. The data collected, such as pain and anxiety scores, were taken from subjective scores of patients, and the results obtained were influenced by subjective factors to some extent;
2. The present study is a clinical randomized controlled study. Therefore, a large sample, multicenter randomized controlled study is still needed to confirm the results further.

## 6 Conclusion

In conclusion, compared with PCNL managed by general anesthesia ERAS, PCNL with local anesthesia applied to ERAS management reduces surgical stimulation in patients through multiple pathways, thus reducing surgical stress and promoting recovery without compromising surgical efficacy and safety. Preliminarily, using local anesthesia for ERAS-managed PCNL is feasible and can benefit patients more.

## Data Availability

The original contributions presented in the study are included in the article/Supplementary Material, further inquiries can be directed to the corresponding authors.
